# Review of the Chemical Composition, Pharmacological Effects, Pharmacokinetics, and Quality Control of *Boswellia carterii*

**DOI:** 10.1155/2022/6627104

**Published:** 2022-01-13

**Authors:** Kai Huang, Yanrong Chen, Kaiyong Liang, Xiaoyan Xu, Jing Jiang, Menghua Liu, Fenghua Zhou

**Affiliations:** ^1^School of Traditional Chinese Medicine, Southern Medical University, Guangzhou 510515, China; ^2^First Clinical Medical College, Southern Medical University, Guangzhou 510515, China; ^3^Second Clinical Medical College, Southern Medical University, Guangzhou 510515, China; ^4^School of Pharmaceutical Sciences, Southern Medical University, Guangzhou 510515, China

## Abstract

**Objective:**

This review aimed to systematically summarize studies that investigated the bioactivities of compounds and extracts from Boswellia.

**Methods:**

A literature review on the pharmacological properties and phytochemicals of *B. carterii* was performed. The information was retrieved from secondary databases such as PubMed, Chemical Abstracts Services (SciFinder), Google Scholar, and ScienceDirect.

**Results:**

The various *Boswellia* extracts and compounds demonstrated pharmacological properties, such as anti-inflammatory, antitumour, and antioxidant activities. B. *carterii* exhibited a positive effect on the treatment and prevention of many ageing diseases, such as diabetes, cancer, cardiovascular disease, and neurodegenerative diseases.

**Conclusion:**

Here, we highlight the pharmacological properties and phytochemicals of *B. carterii* and propose further evidence-based research on plant-derived remedies and compounds.

## 1. Introduction

Frankincense resin comes from the tree of the genus *Boswellia* (family Burseraceae). *Boswellia* resins are recorded in texts with their traditional medical practices in an ancient civilization such as ancient China, Persia, and India. It was subsequently included in Chinese Pharmacopoeia Volume I. *B. carterii* was firstly used as a traditional Chinese medicine for treating urticaria. Modern pharmacological studies confirmed that *B. carterii* could be not only anti-inflammation, antioxidation, antiviral, antimalarial, and antitumour, but also protect liver and nerve. 3-O-Acetyl-11-keto-*β*-boswellic acid, 3*α*-acetoxy-8,24-dienetirucallic acid, and 3*α*-acetoxy-7,24-dienetirucallic acid are related to its anti-inflammatory effect. Incensole acetate plays an important role in its neuroprotective effect.

According to previous comments and reports [1–4], volatile oils and terpenes are the main components of *B. carterii*. However, although many chemical components have been isolated and identified from *B. carterii*, the toxicology and pharmacokinetic studies of *Boswellia* long-term use are lacking. Some review articles on *B. carterii* have been published, mainly concerning its chemical composition and pharmacological activity [1, 5–9]. In this review, we strictly analyze the current state of knowledge of phytochemistry, quality control, pharmacological effects, and pharmacokinetics. It is hoped that this review will fill the knowledge gap, complement the published review on its chemical composition and pharmacological activity, and provide support and perspectives on future research and clinical application of *B. carterii*.

## 2. Methods

A literature search was performed to collect relevant information of the traditional uses, as well as pharmacological properties and phytochemicals of *B. carterii*. Electronic databases were searched, including Google Scholar, SciFinder, PubMed, and ScienceDirect, and several literature articles published before August 2019 were reviewed. Additional primary data such as books were examined, including “The Compendium of Materia Medica” and “Chinese Pharmacopoeia.” Searching for relevant information on *B. carterii* was performed using multiple keywords, such as “*B. carterii*”; “Traditional uses”; “Phytochemistry”; “Pharmacological activities”; “Anti-tumour”; “Anti-inflammatory”; “Wound-healing properties”; and “Hepatoprotective.” All chemical structures were drawn using ChemBioDraw Ultra 14.0 software.

### 2.1. Phytochemistry

The chemical structure of *B. carterii* is primarily composed of terpenoids. A total of 304 compounds were identified, including 148 triterpenes, 94 diterpenes, and 62 compounds classified as volatile oils. All identified compounds are listed and numbered in Table 1.

#### 2.1.1. Volatile Oil

**Scheme 1:**
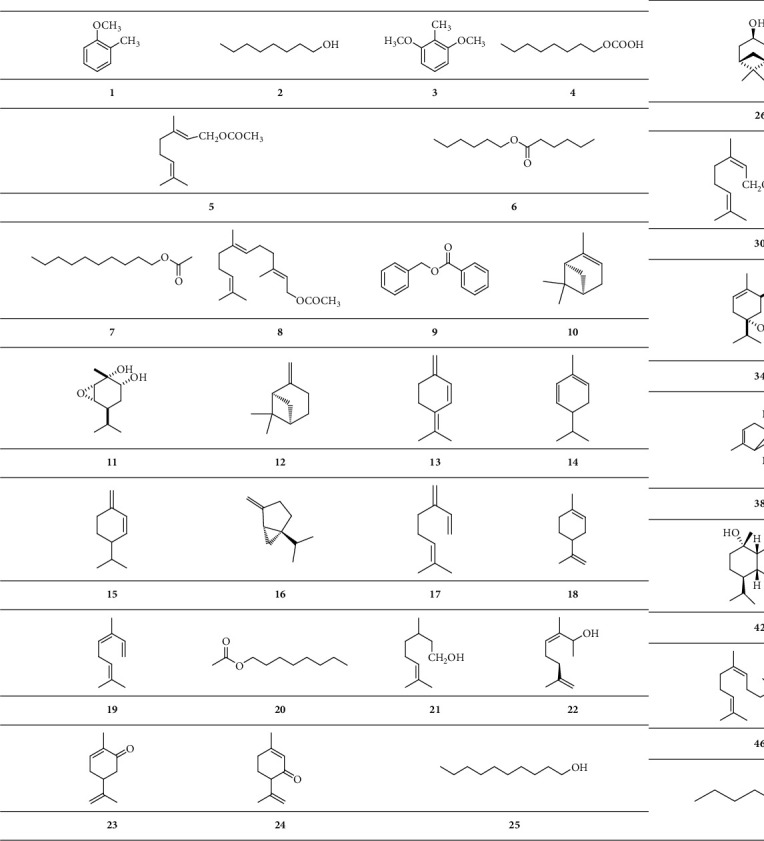
Chemical structural formula of volatile oil.

Volatile oil, also known as an essential oil, is a general term for a class of oily compounds with aromatic odors. It can volatilize at average temperature and can be distilled with water vapor. Volatile oil from *B. carterii* primarily contains monoterpenes, sesquiterpenes, and ester compounds. It is worth mentioning that the classification here does not contain volatile diterpenoids and triterpenes, and we have described them in the corresponding classification (Scheme 1).

#### 2.1.2. Diterpenoid

Diterpenoid refers to a group of compounds whose molecular skeleton contains four isoprene units and 20 carbon atoms.

It contains monocyclic diterpenoids, dicyclic diterpenoids, tricyclic diterpenoids, and tetracyclic diterpenoids. Fifty-one kinds of monocyclic diterpenoids, eighteen kinds of dicyclic diterpenoids, twenty-two kinds of tricyclic diterpenoids, and three kinds of tetracyclic diterpenoids were extracted from *B. carterii*.

**Scheme 2:**
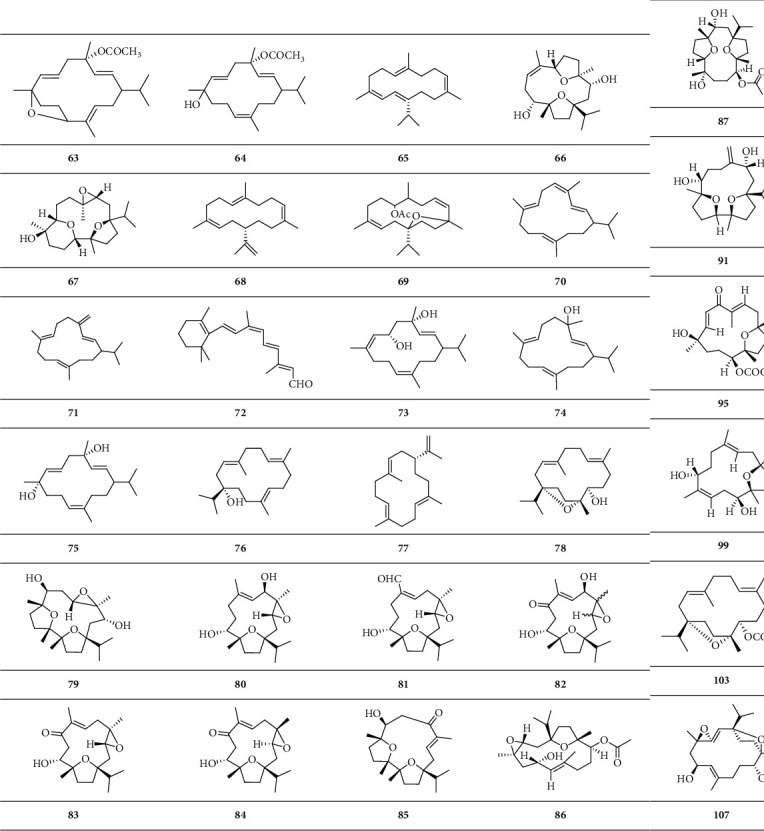
Chemical structural formula of monocyclic diterpenoid.

*(1) Monocyclic Diterpenoid*. Monocyclic diterpenoid is a group of diterpenoids with one closed-loop carbon atom (Scheme 2).

**Scheme 3:**
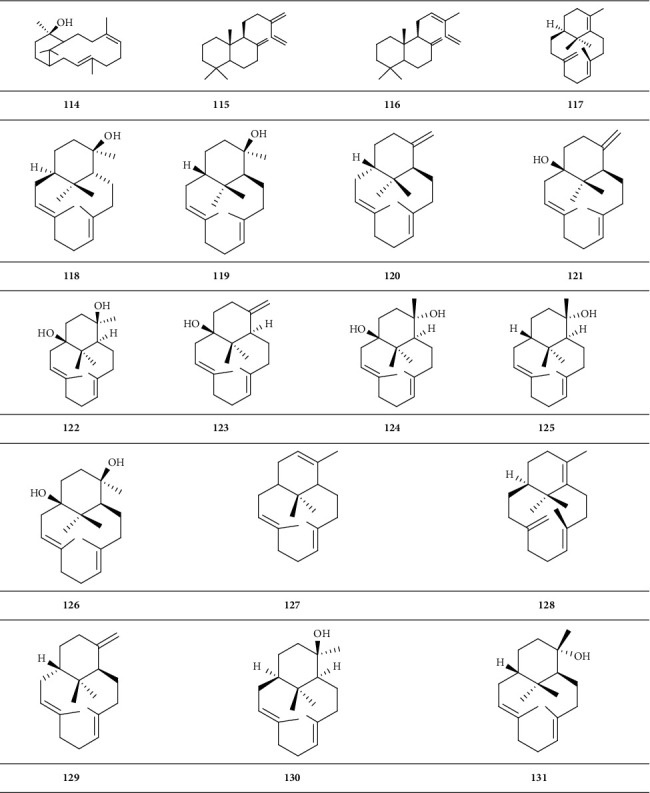
Chemical structural formula of dicyclic diterpenoid.

*(2) Dicyclic Diterpenoid*. Dicyclic diterpenoid is a group of diterpenoids with two closed-loop carbon atoms (Scheme 3).

**Scheme 4:**
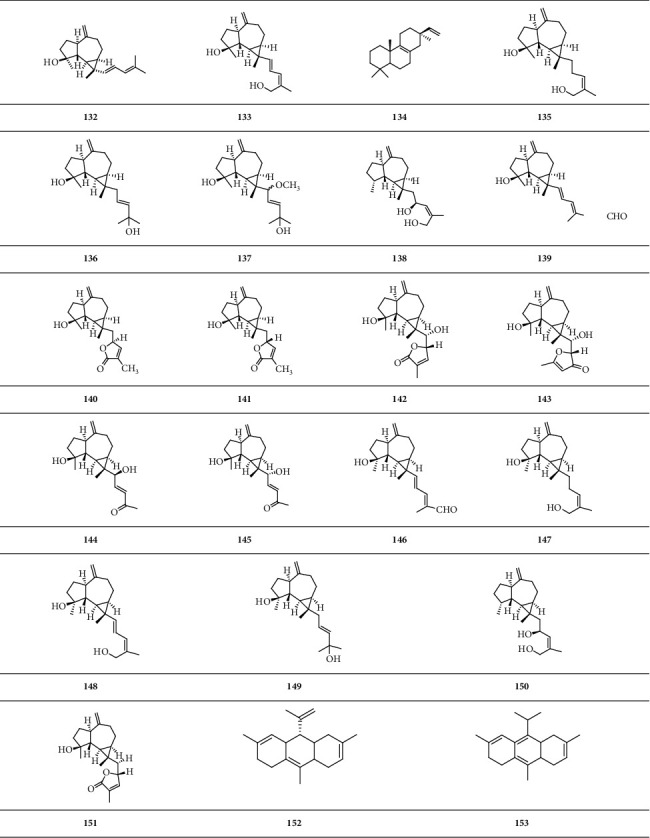
Chemical structural formula of tricyclic diterpenoid.

*(3) Tricyclic Diterpenoid*. Tricyclic diterpenoid is a group of diterpenoids with three closed-loop carbon atoms (Scheme 4).

**Scheme 5:**
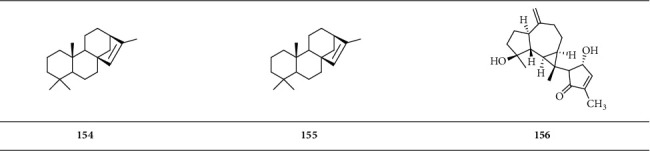
Chemical structural formula of tetracyclic diterpenoid.

*(4) Tetracyclic Diterpenoid*. Tetracyclic diterpenoid is a group of diterpenoids with four closed-loop carbon atoms (Scheme 5).

#### 2.1.3. Triterpenoid

The triterpenoid is a terpenoid composed of 30 carbon atoms. According to the “Isoprene Rule,” most triterpenes consist of the condensation of 6 isoprene units (30 carbons). It can be divided into tetracyclic triterpenoids and pentacyclic triterpenoids. Fifty-seven tetracyclic triterpenes and ninety-one pentacyclic triterpenes were identified from *B. carterii*.

**Scheme 6:**
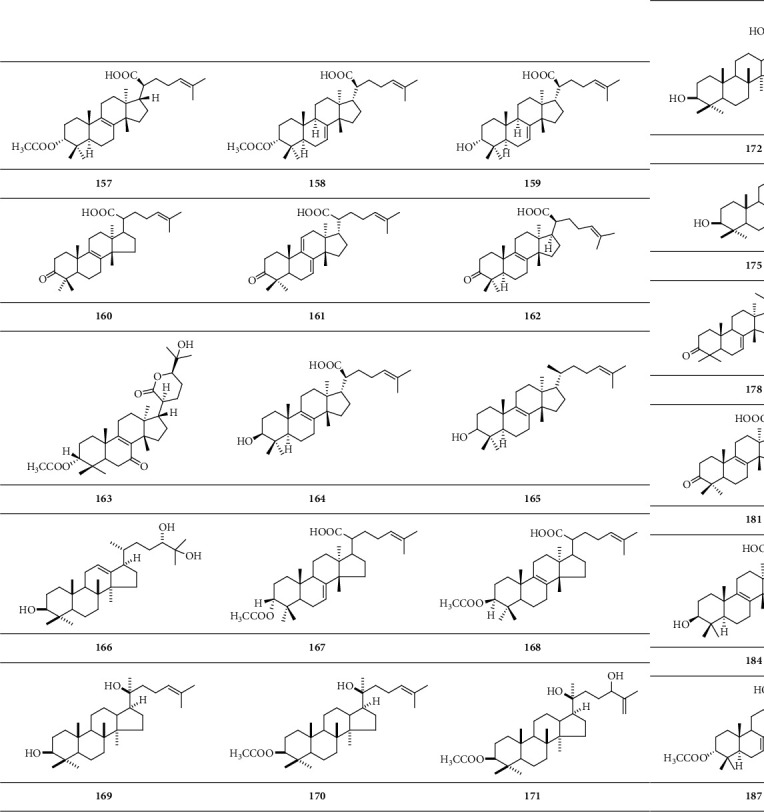
Chemical structural formula of tetracyclic triterpenoid.

*(1) Tetracyclic Triterpenoid*. Tetracyclic triterpenoid is a group of triterpenoids with four closed-loop carbon atoms (Scheme 6).

**Scheme 7:**
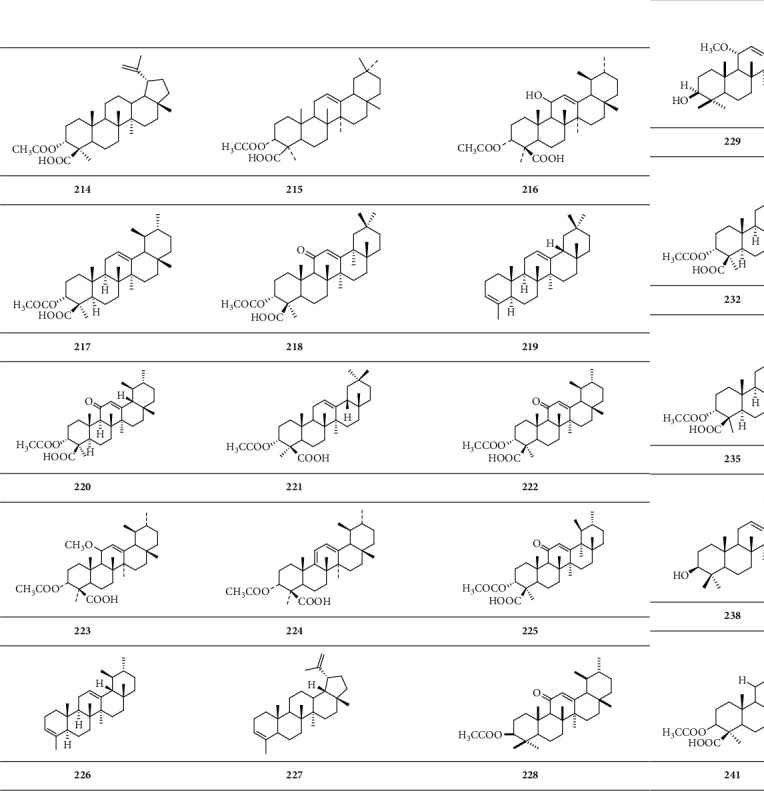
Chemical structural formula of pentacyclic triterpenoid.

*(2) Pentacyclic Triterpene*. A pentacyclic triterpenoid is a group of triterpenoids with five closed-loop carbon atoms (Scheme 7).

## 3. Quality Control

It is vital that quality control is for the safety and effectiveness of traditional Chinese medicine (TCM). Many rapid, sensitive, and stable technologies have been applied for quality analysis of *B. carterii*. A thin-layer chromatography method is developed to differentiate and identify three crucial *Boswellia* species [11]. A total of twenty compounds, which contained two tricyclic diterpenes, twelve triterpenes, and six volatile oil, were detected by GC, GC/MS, SPME, TRSDMC [47], TLC, and HPLC. We summarized the information in Table 2.

## 4. Pharmacology

*B. carterii* has acted as an ethnodrug for a long history because of its pharmacological effects. *Boswellia* contains biologically active compounds that exhibit pharmacological activities (Table 3).

### 4.1. Anti-Inflammatory Effects

It was recorded that *B. carterii* resin has been applied to treat various inflammatory diseases such as rheumatoid arthritis. Boswellic acids, the most well-known active components of *B. carterii* resin, were identified to have anti-inflammatory properties. Boswellic acids, in particular 3-O-acetyl-11-keto-*β*-boswellic acid, interfered with COX-1 and could regulate the anti-inflammatory effect in the way of inhibiting the expression of 5-lipoxygenases (5-LO) and 12-lipoxygenases (12-LO) and the suppression of cyclooxygenases, especially COX-1 [77]. 3-O-Acetyl-11-keto-*β*-boswellic acid reduced Th17 differentiation by interrupting IL-1*ß*-mediated IRAK1 signal, which may regulate IL-1*ß* signal by inhibiting the phosphorylation of IL-1 receptor-related kinase 1 and STAT3 [73].

Microsomal prostaglandin E2 synthase-1 (mPGES-1) was confirmed to be a boswellic acid-interacting protein, and boswellic acid inhibited mPGES-1-mediated prostaglandin (PG) H2 conversion to PGE2 [35]. Besides boswellic acids, other known triterpene acids, particularly 3*α*-acetoxy-8,24-dienetirucallic acid, and 3*α*-acetoxy-7,24-dienetirucallic acid, isolated from *B. carterii* suppressed mPGES-1 [28]. The pull-down experiments and selective inhibition of the expression of iNOS induced by LPS suggested that *ß*-boswellic acid could be anti-inflammation through inhibiting LPS activity [41]. Incensole acetate inhibited cytokine secretion and LPS-induced NF-*κ*B activation through suppressing I*κ*B kinase (IKK) phosphorylation [51]. Incensole acetate reduced the activation of glial cells, the expression of TGF-*β*, IL-1*β*, and TNF-*α* mRNA, and the activation of NF-*κ*B. Incensole acetate induced macrophages dead in closed head injury mice [52]. The above studies indicate that incensole acetate could inhibit inflammation and protect neurons and may show potential effects against ischemia and reperfusion. Furthermore, 3*α*-acetoxy-28-hydroxy-lup-20(29)-en-4*β*-oic acid inhibited the biosynthesis of COX-, 5-LO-, and 12-LO-derived eicosanoids, acting as an efficient inhibitor of cPLA2*α,* and consequently suppressed eicosanoid biosynthesis in intact cells [42].

### 4.2. Antioxidant Effects

Research on the antioxidative effects of *B. carterii* has focused on the compounds 3-O-acetyl-9, 11-dehydro-*β*-mastic acid [28] and alcohol extracts [56]. The antioxidative effects were observed by inhibiting 5-lipoxygenase [28], scavenging oxygen free radicals [78], and inhibiting a significant increase in the lipid peroxidation marker malondialdehyde (MDA) [56]. Besides, the extracts from *B. carterii* showed antioxidant effects using the DPPH- and ABTS-free radical scavenging methods [79]. Interestingly, the methanol fraction of the mastic-containing complex showed anti-inflammatory and antioxidant effects and promoted angiogenesis and epithelial regeneration in mice that had epithelial damage [79]. Oxidative damage is one of the causes of human ageing, and the antioxidant effect of frankincense helps to slow down this process.

### 4.3. Antitumour Effects

*B. carterii* compounds and extracts showed adverse effects on glioblastoma, prostate cancer, fibrosarcoma, neuroblastoma, bladder cancer, leukemia, colon cancer, breast cancer, and liver cancer, which are partly related to the ageing [31, 57, 59, 60]. The cellular pathways modulated by *B. carterii* to exert anticancer effects are involved in the following aspects. *B. carterii* regulated the p21/FOXM1/cyclin B1 pathway, downregulated Aurora B, and upregulated the p53 signalling pathway [57]. Acetyl-lupeolic acid primarily inhibited Akt by directly binding the pleckstrin homology domain. Acetyl-lupeolic acid could lead to three results, namely, the loss of mitochondrial membrane potential, the hindrance of phosphorylation of following targets of the Akt pathway, and the inhibition of the mTOR target p70 ribosomal hexaprotein kinase and *β*-catenin, p65/NF-κB, and c-Myc [59]. *B. carterii* was also shown to significantly inhibit c-Myc expression [80] and block Sp1 DNA-binding activity to inhibit Sp1-stimulated androgen receptor promoter activity [65]. At both Ser473 and Thr308 positions, 3-acetyl-11-keto-*β*-boswellic acid induced Akt phosphorylation [66]. Tirucallic acids functioned in combination with the pleckstrin homeodomain of Akt to inhibit Akt activation and downregulate the pathway that activates Akt [22]. *B. carterii* diterpenoids selectively docked with HIV-1 reverse transcriptase [81]. *B. carterii* triterpenoids target cancer-related proteins, including poly (ADP-ribose) polymerase-1, tankyrase, and the folate receptor [81]. *ß*-Boswellic acid could target cancer-associated proteins, such as proteasomes, 14-3-3 proteins, heat shock proteins, and ribosomal proteins [82]. *B. carterii* essential oil activated heat shock proteins and histone core proteins [61].

Clinically, the combination of *B. carterii*, betaine, and inositol could reduce breast density, relieve pain in benign breast masses, reduce anxiety, and reduce masses in menopausal women [83–85]. Besides, *B. carterii* prolonged the survival of patients with lung cancer [86], reduced fatigue, enhanced vitality, and reduced insulin use in patients with pancreatic cancer [87]. *B. carterii* also exhibited a beneficial effect for patients with bilateral lung and metastatic bladder cancers [88].

### 4.4. Antiviral Effects

The *n*-hexane-soluble mixture, MeOH extract, EtOAc-soluble mixture, *n*-BuOH-soluble mixture, water extract, and H_2_O-soluble mixture of *B. carterii* showed an antiviral effect by inhibiting the hepatitis C virus protease [67] and the Epstein–Barr virus early antigen [21].

### 4.5. Antimicrobial Effects

An antimicrobial effect of *B. carterii* for bacteria (Gram-positive and Gram-negative) and fungi was associated with its essential oils and its smoke [68, 69, 89, 90].

### 4.6. Neuroprotective Effects

A neuroprotective effect has been associated with *B. carterii* extracts that demonstrated antidepressant properties, resistance to inflammation caused by cerebral ischemia, promotion of neurodevelopment, and resistance to Alzheimer's disease [81]. Research in this area has focused on incensole acetate and gum resin from *Boswellia*. The TPRV3 pathway was associated with the antidepressant effect of *B. carterii* [52, 70]. The ability of *B. carterii* to promote nerve development may be related to its ability to increase CaMKII mRNA expression [71]. Incensole acetate reduced NF-*κ*B activity, and GFAP expression in the brain [53] showed an antidepressant effect in acute and chronic treatment cases [91] and reduced the inflammatory response of nerve tissues via the NF-*κ*B pathway [52]. Also, triterpene acids showed cytotoxicity in neuroblastoma [21].

### 4.7. Hepatoprotective Effects

The compounds of *B. carterii* showed a liver protective effect by inhibiting damage from D-galactosamine to HL-7702 cells [4, 19, 24].

### 4.8. Kidney Protective Effects

Prophylactic treatments using *B. carterii* showed benefits in anti-acute and anti-chronic renal failure cases. Oral administration of *B. carterii* induced a reduction in serum creatinine, serum urea, blood urea nitrogen, and C-reactive protein activity [72].

### 4.9. Immunomodulatory Effects

The compounds and fractions of *B. carterii* promoted the transformation of peripheral blood lymphocytes, regulated the expression of lymphokines in mouse spleen cells, dose-dependently inhibited the expression of Th1 cytokines, and dose-dependently promoted the expression of Th2 cytokines [37]. Furthermore, acetyl-11-keto-*β*-boswellic acid, by preventing IL-1R-related kinase 1 phosphorylation and subsequently inhibiting STAT3 phosphorylation, affected the IL-1*β* signalling, thereby inhibiting Th17 cell differentiation [73]. Moreover, it is interesting that the purified compounds showed carrier-dependent immunomodulation in vitro and that the purified compounds are less active than the total compounds [12].

### 4.10. Other Effects

*B. carterii* compounds showed an effect on the lung cell structure of rats [74], affected the development of Callosobruchus species by increasing oxidative stress [47], and reduced the level of oxidation to promote cardiovascular protection [56].

### 4.11. Side Effects

The side effects refer to the pharmacological effects of a drug beyond its therapeutic purpose following the application of a therapeutic amount of the drug. Understanding drug side effects is required to formulate a clinical medication plan and to avoid health risks. The side effects of *B. carterii* are primarily related to smoke-induced reproductive toxicity. Histopathological sections and ultrastructure of the testis and epididymis showed adverse effects on sperm development. Sperm counts, viability, and speed decreased in varying degrees, and the proportion of abnormal sperm increased. Fructose levels in epididymal fluid and prostate fluid were reduced, and also, a luteinizing hormone, testosterone, and follicle-stimulating hormone levels in plasma and protein were reduced [75, 92]. Other studies have shown that sialic acid and carnitine in cauda epididymal plasma are reduced [76].

## 5. Pharmacokinetics

Pharmacokinetics offers scientific support for the clinical use of *B. carterii*. The experiments have shown that 3-acetyl-11-keto-*β*-boswellic acid and 11-keto-*β*-boswellic acid are absorbed more by laboratory animals when administered in processed frankincense forms. Using HPLC, the Cmax of 3-acetyl-11-keto-*β*-boswellic acid and 11-keto-*β*-boswellic acid was 3.197 *μ*g/mL and 2.037 *μ*g/mL for vinegar-processed frankincense (VPF), respectively, and 0.987 *μ*g/mL and 1.937 *μ*g/mL for frankincense oral administration (FRA), respectively [36]. The processed and nonprocessed products exhibited a significant difference in absorption. Meanwhile, 3-acetyl-11-keto-*β*-boswellic acid was absorbed more easily than 11-keto-*β*-boswellic acid, and the values of Cmax were observed in the order of VPF > SFF (stir-fried frankincense) > FRA. The levels of plasma 11-keto-*β*-boswellic acid and 3-acetyl-11-keto-*β*-boswellic acid reduced slowly, especially for the VPF group compared with the FRA group. In the VPF group, pharmacokinetic parameters of 11-keto-*β*-boswellic acid and 3-acetyl-11-keto-*β*-boswellic acid, such as *C*_max_, AUC_0-t_, and AUC_0–∞_, were greatly increased, while V/F and CL/F values were decreased [36]. These results show that the clinical use value of frankincense can be further enhanced [36].

## 6. Discussion

The resins of *B. carterii* have been used for the treatment of inflammation-related diseases, such as traumatic injury and inflammatory pain in China for a long time. Recently, the traditional medicine had become a hot research topic, while more positive effects and other potential medical values have been found. In this study, we listed the isolated components of *Boswellia* resin by category according to previous research and summarized their pharmacological effect on a different model. The different components of *Boswellia* resin have found a series of beneficial effects on many diseases when applied in laboratory research, and some have been approved for clinical use. We hope more research about quality control, and the novel component can be conducted in the future.

## 7. Conclusion

This article reviewed the research performed on the components of *B. carterii* in terms of quality control, phytochemistry, pharmacological effects (including side effects), and pharmacokinetics. We highlighted studies showing that frankincense exhibits anti-inflammatory, antitumour, and antioxidant activities, including some important organ-protective effects on the heart, liver, and kidney. We also found that *B. carterii* exhibits a good effect on the treatment and prevention of geriatric diseases. The review also presented studies showing that pure compounds could exhibit lower immunomodulatory activities than the crude extract, with some progress being made in identifying the mechanisms involved. However, we found that some studies did not investigate relevant toxicology and pharmacokinetic aspects.

Furthermore, the studies did not provide an in-depth evaluation of the bioactivity of the extracts and the isolated compounds, or *in vivo* experiments that might indicate therapeutic relevance. Based on the above research and deficiencies, clinicians should remain cautious when using this plant as a therapeutic drug until further research demonstrates the safety, quality, and efficacy of *B. carterii*. As such, extensive pharmacological and chemical experiments, including human metabolism studies, require future investigations.

## Figures and Tables

**Table 1:** Compounds identified from Boswellia carterii.

Compounds	No.	Reference
*Volatile oil*		
*o*-Methyl anisole	**1**	[2]
Octanol	**2**	[2]
2,6-Dimethoxy toluene	**3**	[2]
Octyl formate	**4**	[2]
Geranyl acetate	**5**	[2]
Hexyl hexanoate	**6**	[2]
Decyl acetate	**7**	[2]
Farnesyl acetate (E, E)	**8**	[2]
Benzyl benzoate	**9**	[2]
*α*-Pinene	**10**	[2]
Olibanumol A	**11**	[10]
*β*-Pinene	**12**	[2]
Isoterpinolene	**13**	[2]
*α*-Phellandrene	**14**	[2]
*β*-Phellandrene	**15**	[2]
Sabinene	**16**	[2]
*β*-Myrcene	**17**	[2]
*d*-Limonene	**18**	[2]
*cis*-Ocimene	**19**	[2]
Octyl acetate	**20**	[2]
*β*-Citronellol	**21**	[2]
*cis*-Carveol	**22**	[2]
Carvone	**23**	[2]
Piperitone	**24**	[2]
1-Decanol	**25**	[2]
Isopinocampheol	**26**	[2]
Bornyl acetate	**27**	[2]
*trans*-Terpin	**28**	[2]
Citronellyl acetate	**29**	[2]
Neryl acetate	**30**	[2]
Olibanumol B	**31**	[10]
Olibanumol C	**32**	[10]
3,6-Dihydroxy-*p*-menth-1-ene	**33**	[10]
*p*-Menth-1-en-4a,6b-diol	**34**	[10]
(-)-*trans*-Sobrerol	**35**	[10]
*p*-Menth-4-en-1,2-diol	**36**	[10]
*p*-Menth-5-en-1,2-diol	**37**	[10]
*α*-Copaene	**38**	[2]
*δ*-Selinene	**39**	[2]
Maaliene	**40**	[2]
Viridiflorol	**41**	[2]
*α*-Muurolol	**42**	[2]
*β*-Bisabolene	**43**	[2]
*cis*-Calamenene (1S)	**44**	[2]
Spathulenol	**45**	[2]
*cis*-Nerolidol	**46**	[2]
*β*-Caryophyllene oxide	**47**	[11]
Palmitic acid	**48**	[12]
1-Hexanol	**49**	[3]
3,5-Dimethoxytoluene	**50**	[3]
Chrysanthenone	**51**	[3]
*cis*-Verbenol	**52**	[3]
Hexyl acetate	**53**	[3]
Linalool	**54**	[3]
Myrtenal	**55**	[3]
Terpinene-4-ol	**56**	[3]
*trans*-Pinocarveol	**57**	[3]
*trans*-Verbenol	**58**	[3]
Z-*β*-Ocimene	**59**	[3]
*α*-Pinene epoxide	**60**	[3]
*β*-Bourbonene	**61**	[3]
*β*-Thujone	**62**	[3]

*Monocyclic diterpenoid*		
Duva-3,9,13-triene-1 *α*-hydroxy-5,8-oxide-1-acetate	**63**	[2]
Duva-3,9,13-triene-1,5 *α*-diol-1-acetate	**64**	[2]
Cembrene C	**65**	[13]
Boscartin A	**66**	[14]
Boscartin P	**67**	[4]
Cembrene A	**68**	[13]
Isoincensole acetate	**69**	[13]
Cembrene	**70**	[2]
Isocembrene	**71**	[2]
9-*cis*-Retinal	**72**	[2]
Duva-4,8,13-triene-1,3 *α*-diol	**73**	[2]
Thunbergol	**74**	[2]
Duva-3,9,13-triene-1,5 *α*-diol	**75**	[2]
Serratol	**76**	[11]
Cembrene A	**77**	[15]
Incensole	**78**	[15]
Boscartin B	**79**	[14]
Boscartin C	**80**	[14]
Boscartin D	**81**	[14]
Boscartin E	**82**	[14]
Boscartin F	**83**	[14]
Boscartin G	**84**	[14]
Boscartin H	**85**	[14]
Boscartin Q	**86**	[4]
Boscartin R	**87**	[4]
Boscartin S	**88**	[4]
Boscartin T	**89**	[4]
Boscartin U	**90**	[4]
Boscartin V	**91**	[4]
Boscartin W	**92**	[4]
Boscartin X	**93**	[4]
Boscartin Y	**94**	[4]
Boscartin Z	**95**	[4]
Boscartin AA	**96**	[4]
Boscartin AB	**97**	[4]
Boscartin AC	**98**	[4]
Boscartin AD	**99**	[4]
Boscartin AE	**100**	[4]
Boscartin AF	**101**	[4]
Boscartin AG	**102**	[4]
Incensole acetate	**103**	[15]
Incensole oxide	**104**	[14]
(rel)-(1S,5 R,7E,11 E)-1-Isopropyl-8,12-dimethyl-4-methylenecyclotetradeca-7,11-diene-1,5-diol	**105**	[16]
1,4-Epoxy-8,13-cembrandien-5,12-diol	**106**	[16]
Boscartin C	**107**	[16]
Boscartin E	**108**	[16]
Boscartin I	**109**	[16]
Boscartin J	**110**	[16]
Boscartin K	**111**	[16]
Myrcene	**112**	[17]
Δ-3-Carene	**113**	[17]

*Dicyclic diterpenoid*		
Verticiol	**114**	[2]
Sclarene	**115**	[2]
Naphthalene decahydro-1,1,4a-trimethyl-6-methylene-5-(3-methyl-2-pentenyl)	**116**	[2]
Verticilla-4(20),7,11-triene	**117**	[15]
(-)-Limonene	**118**	[17]
(R)-Linalool	**119**	[17]
1,8-Cineole	**120**	[17]
1-Octanol	**121**	[17]
*ent*-13-epi-Verticillanediol	**122**	[3]
*ent*-Isoverticillenol	**123**	[3]
*ent*-Verticillanediol	**124**	[3]
*ent*-Verticillol	**125**	[3]
*E*-*β*-Ocimene	**126**	[17]
Isoverticillene	**127**	[3]
*p*-Cymene	**128**	[17]
Verticillene	**129**	[3]
Verticillol	**130**	[3]
*α*-Terpineol	**131**	[17]

*Tricyclic diterpenoid*		
Olibanumol D	**132**	[18]
Boscartol A	**133**	[19]
Phenanthrene-7-ethenyl-1,2,3,4,4a,5,6,7,8,9,10,10a-dodecahydro-1,1,4a,7-tetramethyl	**134**	[2]
Boscartol B	**135**	[19]
Boscartol C	**136**	[19]
Boscartol D	**137**	[19]
Boscartol E	**138**	[19]
Boscartol F	**139**	[19]
Boscartol H	**140**	[19]
Boscartol I	**141**	[19]
Boscartol K	**142**	[20]
Boscartol L	**143**	[20]
Boscartol M	**144**	[20]
Boscartol N	**145**	[20]
Boscartol F	**146**	[20]
Boscartol B	**147**	[20]
Boscartol A	**148**	[20]
Boscartol C	**149**	[20]
Boscartol E	**150**	[20]
Boscartol H	**151**	[20]
*α*-Thujene	**152**	[17]
Camphene	**153**	[17]

*Tetracyclic diterpenoid*		
Isophyllocladene (kaur-15-ene)	**154**	[2]
Beyerene	**155**	[2]
Boscartol G	**156**	[19]

*Tetracyclic triterpenoid*		
3*α*-O-Acetyl-8,24-dien-tirucallic acid	**157**	[15]
3*α*-Acetoxytirucalla-7,24-dien-21-oic acid	**158**	[21]
3*α*-Hydroxytirucalla-7,24-dien-21-oic acid	**159**	[21]
3-Oxo-tirucallic acid	**160**	[22]
3-Oxotirucalla-7,9(11),24-trien-21-oic acid	**161**	[23]
3-Oxo-8,24-dien-tirucallic acid	**162**	[11]
Boscartene A	**163**	[24]
3*β*-Hydroxytirucalla-8,24-dien-21-oic acid	**164**	[21]
*α*-Elemolic acid	**165**	[21]
Olibanumol J	**166**	[10]
3-*α*-Acetoxy-tirucallic acid	**167**	[22]
3-*β*-Acetoxy-tirucallic acid	**168**	[22]
Dammarenediol	**169**	[25]
Dammarenediol acetate	**170**	[25]
3-O-Acetyl-3*β*,20S,24-trihydeoxy-dammar-25-ene	**171**	[25]
Isofouquierol	**172**	[25]
Isofouquierol acetate	**173**	[25]
Ocotillol acetate	**174**	[25]
3*β*-Hydroxymansumbin-13(17)-en-16-one	**175**	[25]
Mansumbinol	**176**	[25]
Isomasticadienonic acid	**177**	[26]
Masticadienonic acid	**178**	[26]
3,4-Seco-olean-12-en-3,28-dioic acid	**179**	[26]
3,4-Seco-olean-18-en-3,28-dioic acid	**180**	[26]
Elemonic acid	**181**	[27]
3-*α*-Hydroxy-8,24-dienetirucallic acid	**182**	[28]
3*α*-Acetoxy-8,24-dienetirucallic acid	**183**	[28]
3-*β*-Hydroxy-8,24-dienetirucallic acid	**184**	[28]
3-Oxo-8,24-dienetirucallic acid	**185**	[28]
3-*α*-Hydroxy-7,24-dienetirucallic acid	**186**	[28]
3*α*-Acetoxy-7,24-dienetirucallic acid	**187**	[28]
Roburic acid	**188**	[28]
4, (23)-Dihydroroburic acid	**189**	[28]
4, (23)-Dihydro-11-keto-roburic acid	**190**	[28]
4, (23)-Dihydronyc-tanthic acid	**191**	[28]
Boscartene B	**192**	[24]
Boscartene C	**193**	[24]
Boscartene D	**194**	[24]
Boscartene E	**195**	[24]
Boscartene F	**196**	[24]
Boscartene G	**197**	[24]
Boscartene H	**198**	[24]
Boscartene I	**199**	[24]
Isoflindissone lactone	**200**	[24]
Boscartene J	**201**	[24]
Boscartene K	**202**	[24]
3-Hydroxy-tirucallic acid	**203**	[12]
Boscartene L	**204**	[29]
Boscartene M	**205**	[29]
Boscartene N	**206**	[29]
Trametenolic acid B	**207**	[29]
3-Oxotirucalla-7, 9 (11), 24-trien-21-oic acid	**208**	[29]
(20S)-3,7-Dioxo-tirucalla-8,24-dien-21-oic acid	**209**	[29]
20,21-Dinortirucalla-8,24-diene-3*β*-ol-7-one	**210**	[16]
3-Oxo-tirucalla-8, 24-dien-21-oic acid	**211**	[16]
3*β*-Hydroxytirucalla-8, 24-dien-21-oic acid	**212**	[16]
3*α*-Hydroxytirucalla-8, 24-dien-21-oic acid	**213**	[16]
Pentacyclic triterpene		
Lup-20-ene-3*α*-acetoxy-24-acid	**214**	[30]
*α*-Boswellic acid acetate	**215**	[31]
3-O-Acetyl-11-hydroxy-*β*-boswellic acid	**216**	[28]
*β*-Boswellic acid acetate	**217**	[32]
3*α*-Acetyl-11-keto-*α*-boswellic acid	**218**	[33]
24-Noroleana-3,12-diene	**219**	[34]
3-O-Oxalyl-11-*β*-keto-boswellic acid	**220**	[35]
3-O-Acetyl-*α*-boswellic acid	**221**	[26]
3-Acetyl-11-keto-*β*-boswellic acid	**222**	[36]
3-O-Acetyl-11-methoxy-*β*-boswellic acid	**223**	[28]
3-O-Acetyl-9,11-dehydro-*β*-boswellic acid	**224**	[28]
3*α*-Acetyl-11-keto-*β*-boswellic acid	**225**	[33]
24-Norursa-3,12-diene	**226**	[34]
24-Norlupa-3,20(29)-diene	**227**	[34]
Neoilexonol acetate	**228**	[25]
Triptohypol F	**229**	[18]
Lupenyl formate	**230**	[18]
3-O-Acetyl-11-keto-*β*-boswellic acid	**231**	[11]
*β*-Acetyl-boswellic acid	**232**	[11]
*α*-Acetyl-boswellic acid	**233**	[11]
3-O-Acetyl-lupeolic acid	**234**	[28]
3-O-Acetyl-28-hydroxy-lupeolic acid	**235**	[28]
Acetyl-11-dien-*β*-boswellic acid	**236**	[37]
Acetyl-lupeolic acid	**237**	[38]
*α*-Amyrin	**238**	[39]
3-O-Acetyl-boswellic acid	**239**	[40]
Olibanumol K	**240**	[25]
3-Oxalyl-*β*-boswellic acid	**241**	[41]
Acetyl-hydroxy-lupeolic acid	**242**	[42]
3-O-Acetyl-9,11-dehydro-*β*-boswellic acid	**243**	[43]
9,11-Dehydro-*β*-boswellic acid	**244**	[44]
11-Keto-*α*-boswellic acid	**245**	[27]
3-O-Acetyl-*β*-boswellic acid	**246**	[28]
Acetyl-*β*-boswellic acid	**247**	[45]
11-Keto-*β*-boswellic acid	**248**	[46]
Acetyl-11*α*-methoxy-*β*-boswellic acid	**249**	[21]
Oleanolic acid	**250**	[39]
Betulin	**251**	[39]
Betulinic acid	**252**	[39]
11-Keto-boswellic acid	**253**	[40]
Olibanumol H	**254**	[10]
Olibanumol I	**255**	[10]
Isofpuquierol	**256**	[10]
3-Epi-*α*-amyrin	**257**	[25]
Olibanumol L	**258**	[25]
Olibanumol M	**259**	[25]
Olibanumol N	**260**	[25]
Epilupeol	**261**	[25]
Epilupeol acetate	**262**	[25]
Lup-20(30)-ene-3*α*,29-diol	**263**	[25]
Glochidiol	**264**	[25]
Lupeol	**265**	[25]
Lup-20(29)-ene-2*α*,3*β*-diol	**266**	[25]
3*β*-Acetoxylup-20(29)-en-11*β*-ol	**267**	[25]
Lupenone	**268**	[25]
Urs-9(11),12-dien-3*β*-ol	**269**	[25]
Neoilexonol	**270**	[25]
Urs-12-ene-3*β*,11*α*-diol	**271**	[25]
Urs-12-ene-3*α*,11*α*-diol	**272**	[25]
Olibanumol E	**273**	[18]
Olibanumol F	**274**	[18]
Olibanumol G	**275**	[18]
18H*α*,3*β*,20*β*-ursanediol	**276**	[23]
3-Oxalyl-11-keto-*β*-boswellic acid	**277**	[41]
3-Succinoyl-*β*-boswellic acid	**278**	[41]
3-Succinoyl-11-keto-*β*-boswellic acid	**279**	[41]
3-Glutaroyl-*β*-boswellic acid	**280**	[41]
3-Glutaroyl-11-keto-*β*-boswellic acid	**281**	[41]
3-Carboxymethylenoxy-*β*-boswellic acid	**282**	[41]
3-Carboxymethylenoxy-11-keto-*β*-boswellic acid	**283**	[41]
11-Keto-*β*-boswellic acid	**284**	[11]
*β*-Boswellic acid	**285**	[11]
*α*-Boswellic acid	**286**	[11]
3-O-Acetyl-11-hydroxy-*β*-boswellic acid	**287**	[43]
Acetyl-9,11-dehydro-*β*-boswellic acid	**288**	[44]
Acetyl-9,11-dehydro-*α*-boswellic acid	**289**	[44]
9,11-Dehydro-*α*-boswellic acid	**290**	[44]
Acetyl-lupeolic acid	**291**	[44]
Moronic acid	**292**	[26]
Oleanonic acid	**293**	[26]
Acetyl-*α*-boswellic acid	**294**	[27]
Acetyl-11-keto-*α*-boswellic acid	**295**	[27]
3-Acetyl-9,11-dehydro-*α*-boswellic acid	**296**	[27]
3-Acetyl-9,11-dehydro-*β*-boswellic acid	**297**	[27]
3-O-Acetyl-11-keto-*β*-boswellic acid	**298**	[28]
Lupeolic acid	**299**	[28]
*β*-Boswellic acid	**300**	[45]
*α*-Boswellic acid	**301**	[46]
Acetyl-11-keto-*β*-boswellic acid	**302**	[12]
3-O-Acetyl-11-keto-boswellic acid	**303**	[16]
21*β*-Hydroxy-3-O-acetyl-11-keto-boswellic acid	**304**	[16]

The bold values refer to the relationship corresponding to the chemical structural formula in Schemes 1–7.

**Table 2:** Quantitative analysis for the quality control of *Boswellia carterii*.

Compounds	Method	Result	Reference
Acetyl-11-keto-*β*-boswellic acid Acetyl-*β*-boswellic acid *β*-Boswellic acid *α*-Boswellic acid Acetyl-*α*-boswellic acid 11-Keto-*β*-boswellic acid Acetyl-lupeolic acid Lupeolic acid Acetyl-9,11-dehydro-*α*-boswellic acid 9,11-Dehydro-*α*-boswellic acid Acetyl-9,11-dehydro-*β*-boswellic acid 9,11-Dehydro-*β*-boswellic acid	HPLC	The contents of acetyl-11-keto-*β*-boswellic acid, acetyl-*β*-boswellic acid, *ß*-boswellic acid, *α*-boswellic acid, acetyl-*α*-boswellic acid, 11-keto-*β*-boswellic acid, acetyl-lupeolic acid, lupeolic acid, acetyl-9,11-dehydro-*α*-boswellic acid, 9,11-dehydro-*α*-boswellic acid, acetyl-9,11-dehydro-*β*-boswellic acid, and 9,11-dehydro-*β*-boswellic acid were 40.0, 39.8, 37.2, 26.9, 21.1, 10.1, 7.8, 2.3, 0.28, 0.15, 0.06, and 0.04 mg/g, respectively	[44]
*α*-Thujene *α*-Pinene *α*-Phellandrene	GC-MS	The *α*-thujene (69.16%), *α*-pinene (7.20%), and *α*-phellandrene (6.78%) were the major components of tested essential oil by GC-MS analysis	[47]
*α*-Pinene	GC/MS, UV	*B. carterii* can be distinguished from *B. scar* by comparing optical rotation and chirality. However, storage time and storage conditions increase the variability of the *α*-pinene content, which is related to its optical rotation	[48]
*α*-Pinene *α*-Phellandrene Sabinene Bornyl acetate	GC/MS	The contents of *α*-pinene, *α*-phellandrene, sabinene, and bornyl acetate were 3.11%, 0.03%, 0.26%, and 0.09%, respectively	[2]
*α*-Pinene Isoincensole acetate	GC/MS	The fibre coating material, sampling temperature, and sampling time will affect the test results. The polydimethylsiloxane/divinylbenzene (PDMS/DVB) fibre ageing was found as the most effective method to capture the diterpene characteristics of olibanum, with a sampling time of 1 h and a sampling temperature of 80°C. The contents of *α*-pinene and isoincensole acetate in PDMS/DVB fraction were 4.0% and 8.2%, respectively	[13]
*α*-Pinene Isoincensole acetate	GC/MS	The contents of *α*-pinene and isoincensole acetate in CH2Cl2 extraction of *B. carterii* were 3.6% and 40.4%, respectively	
ß-Caryophyllene oxide	TLC	ß-caryophyllene oxide was a significant marker compound of *B. carterii/sacra*	[11]
*α*-Pinene *α*-Thujene Methoxydecane	GC-MS	Environmental and human factors resulted in 42 samples of *B. carterii* essential oil exhibited three different chemotypes	[49]

**Table 3:** Pharmacological effects of *B. carterii*.

Models	Constituent/Extract	Mechanism	Reference
*Anti-inflammatory effects*			
Adjuvant-induced arthritis in Lewis rats	Aqueous acetone extract	The extract significantly decreased arthritic scores, reduced paw oedema, and restrained the expression of TNF-*α* and IL-1*β*	[50]
12-O-Tetradecanoylphorbol-13-acetate(TPA)-induced inflammation in specific pathogen-free female ICR mice	MeOH extract, *n*-hexane-soluble fraction EtOAc-soluble fraction, *n*-BuOH-soluble fraction H_2_O-soluble fraction *β*-Boswellic acid Acetyl-*β*-boswellic acid 11-Keto-*β*-boswellic acid Acetyl-11-keto-*β*-boswellic acid Acetyl-11*α*-methoxy-*β*-boswellic acid 9,11-Dehydro-*β*-boswellic acid Acetyl-9,11-dehydro-*β*-boswellic acid *α*-Boswellic acid Acetyl-*α*-boswellic acid Lupeolic acid Acetyl-lupeolic acid *α*-Elemolic acid Elemonic acid 3*α*-Hydroxytirucalla-7,24-dien-21-oic acid 3*α*-Acetoxytirucalla-7,24-dien-21-oic acid 3*β*-Hydroxytirucalla-8,24-dien-21-oic acid Incensole	The H_2_O-soluble fraction and EtOAc-soluble fraction showed the strongest and the weakest anti-inflammatory effects in the fraction group, respectively. All compounds showed an anti-inflammatory effect	[38]
HeLa cells, 293T cells, RAW 264.7 macrophage cell, Jurkat T leukemia cells, 5.1 Jurkat and HeLa-Tat-Luc cell lines, A549 cells, human peripheral monocytes, female Sabra mice	Incensole acetate (IA) Incensole (IN)	IA and IN (3-280 *μ*M) inhibited I*κ*B*α* degradation. IA inhibited I*κ*B*α* and p65 phosphorylation by impairment of IKK activation and interfered with TAK/TAB-mediated phosphorylation of IKK*α*/*β* activation loop. IA inhibited NF-*κ*B accumulation in cell nuclei and DNA binding, which may be related to its inhibition of gene expression by NF-*κ*B	[51]
LPS-induced inflammatory in rat C6 glioma cell and human peripheral monocytes	Incensole acetate (IA)	100 *μ*mol/L IA, restraining the expression of IL-1b and TNF-*α* mRNA, inhibited the activation and mRNA level of NF-*κ*B in human peripheral blood monocytes and C6 glioma cells	[52]
Lipopolysaccharide-activated mouse peritoneal macrophages	Olibanumol A Olibanumol B Olibanumol C Olibanumol H Olibanumol I 3,6-Dihydroxy-p-menth-1-ene *p*-menth-1-en-4*α*,6*β-*diol(-)-*trans*-sobrerol *p*-menth-4-en-1,2-diol *p*-Menth-5-en-1,2-diol Isofpuquierol Epilupeol	Twelve compounds inhibited the production of NO	[10]
Lipopolysaccharide-activated mouse peritoneal macrophages	Olibanumol D Olibanumol E	Two compounds exhibited nitric oxide production inhibitory activity	[18]
Carrageenan-induced paw oedema and Carrageenan-induced pleurisy in adult male CD1 mice and Wistar Han rats A549 cells and human whole blood	*α*-Amyrin 3-O-Acetyl-*β*-boswellic acid 3-O-Acetyl-11-keto-*β*-boswellic acid *β*-Boswellic acid 11-Keto-*β*-boswellic acid 3-O-Oxalyl-11-*β*-keto-boswellic acid	Human mPGES-1 was identified as one of the *β*-boswellic acid-binding proteins. The boswellic acid is capable of reversibly inhibiting the conversion of prostaglandin (PG) H2 to PGE2, which is mediated by mPGES-1. Besides, in A549 cells, boswellic acids restrained PGE2 generation, and in human whole blood, *β*-boswellic acid diminished PGE2 biosynthesis induced by LPS. *β*-boswellic acid (1 mg/kg) can inhibit pleurisy in rats, accompanied by decreasing levels of PGE2, and can also reduce paw oedema in mice	[35]
Cooperation-induced cerebral ischemic injury in C57BL/6 mice and TRPV 3-deficient mice	Incensole acetate (IA)	0-50 mg/kg IA reduced the levels of TNF-*α*, IL-1*β*, and TGF-*β*, the activity of NF-*κ*B, and the expression of GFAP in the brain of model mice in a dose-dependent manner	[53]
Formalin and carrageenan-induced paw oedema in mice and oxytocin-induced dysmenorrhea in mice	Water extract of frankincense (FWE)	FWE significantly inhibited PGE2 production, and 5.2 g/kg FWE inhibited nitrite production	[54]
Neutrophils, monocytes, and platelets from human blood	Lupeolic acid (LA) Acetyl-lupeolic acid (Ac-LA) Acetyl-hydroxy-lupeolic acid (Ac–OH–LA)	Ac–OH–LA, which may directly hamper with cPLA2a activity (IC50 = 3.6 *μ*M), lowered the biosynthesis of COX-, 5-LO-, and 12-LO-derived eicosanoids, with consistent IC50 value ranging from 2.3 to 6.9 *μ*M.	[42]
A549 cells	3-*α*-Hydroxy-8,24-dienetirucallic acid 3*α*-Acetoxy-8,24-dienetirucallic acid 3-*β*-Hydroxy-8,24-dienetirucallic acid 3-Oxo-8,24-dienetirucallic acid 3-*α*-Hydroxy-7,24-dienetirucallic acid 3*α*-Acetoxy-7,24-dienetirucallic acid Roburic acid 4, (23)-Dihydroroburic acid 4, (23)-Dihydro-11-keto-roburic acid Lupeolic acid 3-O-Acetyl-lupeolic acid 3-O-Acetyl-28-hydroxy-lupeolic acid	Twelve compounds suppressed mPGES-1 with increased potencies. 3*α*-Acetoxy-7,24-dienetirucallic acid and 3*α*-acetoxy-8,24-dienetirucallic acid suppressed mPGES-1 activity with IC50 = 0.4 *μ*M, each	[28]
Xylene-induced ear oedema model and formalin-inflamed hind paw model in Kunming mice	Frankincense oil extract (FOE) *α*-Pinene Linalool 1-Octanol	FOE and three compounds restrained inflammatory infiltrates and COX-2 overexpression induced by the nociceptive stimulus	[55]
LPS-induced NO production in RAW 264.7 cell	Boscartol K Boscartol L Boscartol F	Boscartol K, boscartol L, and boscartol F inhibited NO production.	[20]
LPS-induced NO production in RAW 264.7 cell	(rel)-(1S,5 R,7E,11 E)-1-Isopropyl-8,12-dimethyl-4-methylenecyclotetradeca-7,11-diene-1,5-diol 3-Oxo-tirucalla-8, 24-dien-21-oic acid 3*β*-Hydroxytirucalla-8,24-dien-21-oic acid 3-O-Acetyl-11-keto-boswellic acid	Four compounds restrained NO production with IC50 values of 1.32, 3.04, 1.42, and 3.25 *μ*M, respectively	[16]

*Antioxidant effects*			
5-Lipoxygenase	3-O-Acetyl-9,11-dehydro-*β*-boswellic acid 3-O-Acetyl-11-methoxy-*β*-boswellic acid 9,11-Dehydro-*β*-boswellic acid	Three compounds inhibited 5-LO activity to varying degrees, of which 3-O-acetyl-9,11-dehydro-*β*-boswellic acid almost completely abolished 5-LO activity	[28]
ABTS radical cation	Methanol extract	1000 *μ*g/kg extract exhibited a weak antioxidant activity	[56]

*Antitumour effects*			
The human glioblastoma cells, U251 and U87-MG U87-MG-induced tumour model in BALB/c-nu nude mice	3-O-Acetyl-11-keto-*β*-boswellic acid	3-O-Acetyl-11-keto-*β*-boswellic acid, via the p21/FOXM1/cyclin B1 pathway, stop glioblastoma cells at the G2/M phase, which was related to the inhibition of mitosis through Aurora B/TOP2A pathway and the induction of mitochondrial-dependent apoptosis	[57]
LNCaP and PC-3 cell	Acetyl-keto-*β*-boswellic acid	20 *μ*g/ml acetyl-keto-*β*-boswellic acid induced apoptosis in LNCaP and PC-3 cell via a DR5 regulated pathway, which induced the expression of CAAT/enhancer-binding protein homologous protein	[58]
PC-3 cell MDA-MB-231 cell	Acetyl-lupeolic acid	Directly bound to the pleckstrin homology domain, acetyl-lupeolic acid (0-20 *μ*g/mL) advertised hindrance of phosphorylation of following targets of the Akt signalling pathway and nuclear accumulation of the mTOR target p70 ribosome and p65/NF-*κ*B, *β*-catenin and c-Myc six protein kinase	[59]
B16F10 cell HT-1080 cell	Boswellic acid acetate	In B16F10 cells, boswellic acid acetate (25 *μ*M) inhibited cell migration activity, lured cell differentiation, blocked the cell population in the G1 phase, and restrained topoisomerase II activity. Boswellic acid acetate lured apoptosis of HT-1080 cells and prevented the secretion of MMPs from HT-1080 cells	[60]
Myeloid leukemia cells HL-60, U937, ML-1, erythrocyte leukemia cells DS-19 and K562	BC-4, a mixture contained *α*- and *β*-boswellic acid acetate	In myeloid leukemia cells, BC-4 (24.2 *μ*M) lured monocytic differentiation. BC-4 also increased specific and nonspecific esterases. Besides, BC-4 dose- and time-dependently inhibited growth of all cell lines tested	[31]
IMR-32, NB-39, and SK-N-SH cell	*β*-Boswellic acid, acetyl-*β*-boswellic acid, 11-keto-*β*-boswellic acid, acetyl-11-keto-*β*-boswellic acid, acetyl-11*α*-methoxy-*β*-boswellic acid, 9,11-dehydro-*β*-boswellic acid Acetyl-9,11-dehydro-*β*-boswellic acid, acetyl*α*-boswellic acid, lupeolic acid, acetyl-lupeolic acid, elemonic acid, 3*α*-hydroxytirucalla-7,24-dien-21-oic acid, 3*α*-acetoxytirucalla-7,24-dien-21-oic acid, incensole Incensole acetate	In the above cells, these fifteen compounds exhibited potent cytotoxic activities	[21]
Text of activation of NOR1	Acetyl-9,11-dehydro-*β*-boswellic acid Elemonic acid 3*α*-Hydroxytirucalla-7,24-dien-21-oic acid 3*β*-Acetoxytirucalla-7,24-dien-21-oic acid 3*α*-Hydroxytirucalla-8,24-dien-21-oic acid	Five compounds indicated potent inhibitory effects of the activation of (-/+)-(E)-methyl-2[(E)-hydroxyimino]-5-nitro-6-methoxy-3-hexemide (NOR 1).	[21]
PC-3 cell	3*α*-Acetyl-11-keto-*α*-boswellic acid	3*α*-acetyl-11-keto-*α*-boswellic acid inhibited the proliferation of human PC-3 cells and induced apoptosis, as shown by the activation of caspase-3 and the induction of DNA fragmentation. Furthermore, 3*α*-acetyl-11-keto-*α*-boswellic acid inhibited the proliferation and induced apoptosis of PC-3 xenografted to the chorioallantoic membrane of the chicken chorioallantoic membrane.	[33]
Bladder cancer cell J82 Immortalized normal bladder cell UROtsa	Frankincense essential oil (FEO)	FEO-activated signal of IL-6, histone core proteins, and heat shock proteins. FEO induced selective cancer cell death through NRF-2-mediated oxidative stress.	[61]
Jurkat cell	*Boswellia* water extract	*Boswellia* extract (200 *μ*g/ml) promoted apoptosis of Jurkat cells and stopped cell differentiation in the G1 phase.	[62]
Bladder cancer cell J82	Frankincense oil	Through activating genes responsible for cell apoptosis, cell growth inhibition, and cell cycle arrest, frankincense oil inhibited the cell viability of J82 cells, but cell death did not result in DNA fragmentation.	[63]
N-2A cells	Ethanol fraction of frankincense	Ethanol fraction showed cytotoxicity to neuro-2A cell with LC50 of 0.081 mg/mL.	[64]
Prostate cancer cells LNCaP and PC-3	Acetyl-11-keto-*β*-boswellic acid	Based on the binding activity of Sp1, the active compound downregulated AR short promoter and hindered cellular proliferation. Luring p21 (WAF1/CIP1) and preventing cyclin D1 in cells, the compound (20-40 *μ*M) induced G1 phase cell cycle arrest.	[65]
HT-29, HCT-116, SW480, and LS174 T colon cancer cell lines	3-acetyl-11-keto-*β*-boswellic acid	3-acetyl-11-keto-*β*-boswellic acid (30 *μ*M) could activate the PI3K/Akt pathway. However, when we inhibited the PI3K pathway, the cell apoptosis induced by 3-acetyl-11-keto-*β*-boswellic acid would enhance	[66]
Hep-G2 cell	Verticilla-4(20),7,11-triene	Verticilla-4(20),7,11-triene showed an inhibitory effect against the proliferation of Hep-G2 cell line	[15]
PTEN-overexpressing PC-3 cells Peripheral blood mononuclear cells LNCaP cell PC-3 tumours xenografted to nude mice and chick chorioallantoic membranes	3-Oxo-tirucallic acid 3-*α*-Acetoxy-tirucallic acid 3-*β*-Acetoxy-tirucallic acid	Tirucallic acids inhibited Akt activity, downregulated the pathway of Akt activation, and induced apoptosis in prostate cancer cell lines. However, 3-*β*-acetoxy-tirucallic acid showed no significant activation of Akt1, which lacks the pleckstrin homology domain. The compounds inhibited the proliferation and induced apoptosis of tumours xenografted to the allantoic membrane of chicken veins, and postponed the progression of pre-established prostate tumours in nude mice without causing systemic toxicity	[22]

*Antiviral effects*			
Hepatitis C virus	*Boswellia carterii*	*B. carterii* showed toxicity to the hepatitis C virus with IC50 of 23 mg/mL, which may be related to its inhibition of hepatitis C virus protease.	[67]
TPA-induced production of EBV-EA in Raji cell	*β*-Boswellic acid Lupeolic acid Acetyl-lupeolic acid Elemonic acid 3*α*-Hydroxytirucalla-7,24-dien-21-oic acid 3*α*-Acetoxytirucalla-7,24-dien-21-oic acid 3*β*-Hydroxytirucalla-8,24-dien-21-oic acid	In Raji cells, the above compounds show dose-dependent inhibition of EBV-EA induction induced by TPA	[21]

*Antimicrobial effects*			
*Staphylococcus aureus* (*S. aureus*) ATCC 29213 *S. aureus* ATCC 25923 *S. aureus* ATCC 43866 *S. epidermidis* DSM 3269 *Escherichia coli* (*E. coli*) ATCC 25922 *Pseudomonas aeruginosa* (*P. aeruginosa*) ATCC 9027 *Candida albicans* (*C. albicans*) ATCC 10231 *C. tropicalis* ATCC 13803	Oleo gum resin oil	The antibacterial activity of the oleo gum resin oils from *B. carterii* was identified and found to show antibacterial activity to the above bacterial	[68]
*Trichosporon ovoides*	Essential oil (EO)	EO showed antibacterial activity against trichosporon ovoides with MIC and MIF of 25 *μ*l/ml and 50 *μ*l/ml, respectively	[69]

*Neuroprotective effects*			
The Sabra line mice were selected to be compliant for 10 generations.	Incensole acetate (IA)	IA has shown potent TRPV3 agonists, which caused anti-anxiety-like and anti-depression-like behavioural effects, with changes in c-Fos activation in the brain	[70]
Anterior cerebral artery ligation-induced cerebral ischemic injury in C57BL/6 mice and TRPV 3-deficient mice	Incensole acetate (IA)	0-50 mg/kg IA dose-dependently reduced the cerebral infarction area and the contents of TNF-*α*, IL-1*β*, and TGF-*β* in the brain of the model mice, the activity of NF-*κ*B, and the expression of GFAP in the brain. The behavioural assessment found that IA dose-dependently reduced nerve damage. Interestingly, IA showed only partial neuroprotective effects in TRPV3-deficient mice	[52]
LPS-induced inflammatory in rat C6 glioma cell and human peripheral monocytes	Incensole acetate (IA)	Incensole acetate (100 *μ*mol/L) downregulated NF-*κ*B activation and mRNA level in both human peripheral monocytes and C6 glioma cells. Moreover, it impaired the inflammatory reaction in human peripheral monocytes	[52]
Weight drop device-induced closed head injury in male Sabra mice	Incensole acetate (IA)	IA (50 mg/kg) alleviated inflammation and neurodegeneration in the hippocampus by inhibiting the mRNA level of TNF-*α* and IL-1*β* after closed head injury. Incensole acetate induced a mild hypothermic effect, but it did not affect tissue oedema formation	[52]
HEK293 cells, female Sabra mice, wild-type C57BL/6, and TRPV3(KO) female mice	Incensole acetate (IA)	IA (50 mg/kg) regulated the expression of c-Fos in mice brain areas, including that related to anxiety and depression. IA (500 *μ*M) activated TRPV3 channels as determined by calcium imaging. IA activated a TRPV3 current in HEK293 cells and relieved depression and anxiety in wild-type but not in TRPV3 KO mice	[70]
The mice fed by breast milk which was generated from the Boswellia-fed mice	*B. carterii*	Pregnancy or lactation mother mice receiving *B. carterii* injection upregulated CaMKII mRNA in the hippocampus of offspring, but no significant change in hippocampal CaMKIV mRNA expression	[71]

*Kidney protective effects*			
Oral adenine-induced chronic renal failure model in adult male albino rats ischemia-reperfusion injury-induced acute renal failure model in adult male albino rats	*Boswellia*	Prophylactic oral administration of *Boswellia* decreased serum urea, blood urea nitrogen, and the activity of C-reactive protein	[72]

*Hepatoprotective effects*			
D-galactosamine-induced toxicity in HL-7702 cell	Boscartol A, boscartol B, boscartol C, boscartol E, boscartol F, boscartol H, and boscartol I	Seven compounds (10 *μ*M) reduced cytotoxicity, which may be the basis of its liver protection	[19]
D-galactosamine-induced cytotoxic in HL-7702 cell	Acetyl-*α*-elemolic acid 3*β*-Hydroxytirucalla-8,24-dien-21-oic acid 3*α*-Hydroxytirucalla-8,24-dien-21-oic acid 3*β*-Hydroxy-mansumbin-13(17)-en-16-one	Four compounds reduced cytotoxic and increased the survival rate in cell	[24]
D-galactosamine-induced toxicity in HL-7702	Boscartin P, boscartin U, boscartin V, boscartin W, boscartin X, boscartin Y, boscartin AA, boscartin AB, boscartin AE, boscartin AF, incensole, incensole oxide acetate, incensole oxide, 1,4-epoxy-8,13-cembrandien-5,12-diol, 4,8-epoxy-8,12-cembrandien-5,12-diol	Fifteen compounds (10 *μ*M) showed hepatoprotective effect against HL-7702 cell injury induced by D-galactosamine	[4]

*Immunomodulatory effects*			
Th17 CD4+T cell, Th1, Th2, and Treg cell	Acetyl-11-keto-*β*-boswellic acid	Slightly increasing the differentiation of Th2 and Treg cells, acetyl-11-keto-*β*-boswellic acid (1 or 5 *μ*M) reduced the differentiation of human CD4 (+) T cells. Further, acetyl-11-keto-*β*-boswellic acid reduced IL-17A released from memory Th17 cells triggered by IL-1*β*, which may involve IL-1*β* signalling by inhibiting the phosphorylation of IL-1 receptor-associated kinase 1 and STAT3	[73]
Peripheral blood lymphocytes	Palmitic acid, lupeol, *β*-boswellic acid, 11-keto-*β*-boswellic acid, acetyl-*β*-boswellic acid, acetyl-11-keto-*β*-boswellic acid, acetyl-*α*-boswellic acid, 3-oxo-tirucallic acid, 3-hydroxy-tirucallic acid	Nine compounds promoted the transformation of peripheral blood lymphocytes	[12]
Murine splenocytes	Ethanol extract and sesame oil extract	Using ethanol as a solvent to deliver resin extracts resulted in significant cytotoxicity, which was not seen when ethanol was added alone. In contrast, when delivered by sesame oil solvent, resin extract dose-dependently inhibited TH1 cytokines and dose-dependently enhanced TH2 cytokines	[37]
Wister albino mice	*Boswellia carterii* smoke	The smoke resulted that alveolar capillaries were damaged, neutrophil nucleus contracted, mitochondria swelled and elongated in type 2 lung cells, type 2 lung cells were shed, most microvilli were shed, and leukocyte neutrophils were exuded in the alveolar cavity	[74]

*Other effects*			
Epinephrine hydrochloride and cool water bath-induced acute cold blood model in SD rats	Stir-fried frankincense (SFF) Vinegar-processed frankincense (VPF) Frankincense oral administration (FRA)	Frankincense (2.7 g/kg) presented more anticoagulant function than its processed products. FRA reduced the levels of DD and TAT and increased the content of PGI2. The processing of frankincense resulted in changes in its absorption and pharmacokinetics	[36]
Myeloid leukemia cells HL-60, U937, and ML-1, and erythrocyte leukemia cells DS-19 and K562	Boswellic acid acetate	The compounds advertised a time- and dose-dependent induction and differentiation on myeloid leukemia cells expressed significant pro-apoptotic effects above 15 mg/ml. They also enriched the red blood cell line leukemia cells DS-19 and K562 at the G1 phase	[31]
Jurkat cell	*Boswellia carterii* Birdw. extract	Frankincense extract induced Jurkat cell apoptosis, promoted Jurkat cell apoptosis, and stopped cell differentiation at G1 phase	[74]
Myeloid leukemia cells NB4, SKNO-1, K562, U937, ML-1, and HL-60	Boswellic acid acetate (BAA)	BAA, under the condition of 20 *μ*g/ml for 24 h, decreased cell membrane potential, and p53 mutation did not affect the pro-apoptotic effect of boswellic acid acetate. Also, BCL-2, Bax, and Bcl-X do not participate in the process of BAA-induced cell membrane potential decline	[32]
Jack bean urease	3-O-Acetyl-9,11-dehydro-*β*-boswellic acid 3-O-Acetyl-11-hydroxy-*β*-boswellic acid 3-O-Acetyl-11-keto-*β*-boswellic acid 11-Keto-*β*-boswellic acid	Four compounds presented an inhibitory effect on Jack bean urease with IC50 of 6.27, 9.21, 16.34, and 85.23 *μ*mol/L, respectively. The inhibitory force may be because of the formation of appropriate hydrogen bonds and the hydrophobic interaction between 3-O-acetyl-9,11-dehydro-*β*-boswellic acid and the urease active site	[43]
*Callosobruchus chinensis* (*C. chinensis*) and C. *maculatus*	*B. carterii* essential oil (BEO)	The essential oil showed toxicity to *C. chinensis* with LC50 and LC90 of 0.066 and 0.096 *μ*L/mL, respectively. It expressed the same effect in *C. maculatus* with LC50 and LC90 of 0.050 and 0.075 *μ*L/mL. BEO showed a concentration-dependent inhibitory effect on its spawning, growth, and development behaviour. It was found that the essential oil induced an increase in the levels of ROS, SOD, and CAT in pests. It also decreased the level of GSH and GSH/GSSG	[47]
Wistar male albino rats	Alcohol extract of olibanum	At a concentration of 1,000 *μ*g/kg, the alcohol extract of olibanum, advertising dose-dependence NO-scavenging action, resulted in a marked increase in the serum levels of LDH, AST, and CK-MB, as well as MDA	[56]

*Side effects*			
Male albino rat	Boswellic smoke	Histopathological sections and ultrastructure of the testis showed adverse effects on sperm development. Sperm analysis revealed that sperm counts, viability, and speed decreased in varying degrees, and the proportion of abnormal sperm increased	[75]
Wistar male albino rat	Boswellic smoke	The smoke resulted that fructose levels in epididymal fluid and prostate fluid were decreased. The histopathological sections and morphological analysis of the epididymis showed an adverse effect on sperm development	[75]
Wistar male albino rat	Boswellic smoke	The smoke caused a decrease in follicle-stimulating hormone, luteinizing hormone, testosterone and protein, sialic acid, and carnitine. Also, the smoke resulted in a decrease in sperm count, reduced vitality, and reduced speed. The testicular ultrastructure showed adverse changes to sperm	[76]

## Data Availability

A literature review on the pharmacological properties and phytochemicals of *B. carterii* was performed. The information was retrieved from secondary databases such as PubMed, Chemical Abstracts Services (SciFinder), Google Scholar, and ScienceDirect.
